# A High-Capacity Optical Metro Access Network: Efficiently Recovering Fiber Failures with Robust Switching and Centralized Optical Line Terminal

**DOI:** 10.3390/s24041074

**Published:** 2024-02-07

**Authors:** Rahat Ullah, Sibghat Ullah, Ahmad Almadhor, Hathal Salamah Alwageed, Abdullah A. Al-Atawi, Jianxin Ren, Shuaidong Chen

**Affiliations:** 1 Institute of Optics and Electronics, Nanjing University of Information Science & Technology, Nanjing 210044, China; 2 Jiangsu Key Laboratory for Optoelectronic Detection of Atmosphere and Ocean, Nanjing University of Information Science & Technology, Nanjing 210044, China; 3 Jiangsu International Joint Laboratory on Meterological Photonics and Optoelectronic Detection, Nanjing University of Information Science & Technology, Nanjing 210044, China; 4 School of Electronic Science and Engineering, Southeast University, Nanjing 210096, China; 5 Department of Computer Engineering and Networks, College of Computer and Information Sciences, Jouf University, Sakaka 72388, Saudi Arabia; 6 College of Computer and Information Sciences, Jouf University, Sakaka 72388, Saudi Arabia; 7 Department of Computer Science, Applied College, University of Tabuk, Tabuk 47512, Saudi Arabia

**Keywords:** optical communication, optical metro access network, wavelength division multiplexing, optical ring network, optical multicarrier generation

## Abstract

This study proposes and presents a new central office (CO) for the optical metro access network (OMAN) with an affordable and distinctive switching system. The CO’s foundation is built upon a novel optical multicarrier (OMC) generation technique. This technique provides numerous frequency carriers that are characterized by a high tone-to-noise ratio (TNR) of 40 dB and minimal amplitude excursions. The purpose is to accommodate multiple users at the optical network unit side in the optical metropolitan area network (OMAN). The OMC generation is achieved through a cascaded configuration involving a single phase and two Mach Zehnder modulators without incorporating optical or electrical amplifiers or filters. The proposed OMC is installed in the CO of the OMAN to support the 1.2 Tbps downlink and 600 Gbps uplink transmission, with practical bit error rate (BER) ranges from 10^−3^ to 10^−13^ for the downlink and 10^−6^ to 10^−14^ for the uplink transmission. Furthermore, in the OMAN’s context, optical fiber failure is a main issue. Therefore, we have proposed a possible solution for ensuring uninterrupted communication without any disturbance in various scenarios of main optical fiber failures. This demonstrates how this novel CO can rapidly recover transmission failures through robust switching a and centralized OLT. The proposed system is intended to provide users with a reliable and affordable service while maintaining high-quality transmission rates.

## 1. Introduction

Metro access networks must provide a high data rate and bandwidth to keep pace with escalating bandwidth demands. The demand for triple-play services necessitates using a high number of distributed laser diodes at both the transmitter and receiver. There is growing evidence that an optical metro access network (OMAN) is a viable option for next-generation optical networks due to its low operating costs, big capacity, low latency, and high transmission efficiency [1,2,3]. Similarly, the convergence of wireless and optical technologies will facilitate the provision of large bandwidth for high data rates, improving service quality [4]. Different techniques are introduced to converge both the optical and wireless networks [5,6]. As a meeting point between service providers and operators, OMANs are particularly well suited for adapting to the extreme acceleration of dynamics in optical systems, using efficient components like optical nodes and transponders [7,8]. This means that these networks can quickly adjust to rapid changes in network conditions like traffic volume. Access and conventional convergence layers can be integrated into the metro access layer of the OMAN [9]. One accepted trend in the optical access network is merging metro and access structures under the cost-effectiveness paradigm to support many users [10]. However, new optical line terminals should be designed to support a large number of users. This allows for transparent reach between the metro network and the end-user. However, bridging optical access and metro networks requires a careful estimation of the deployment costs and power budgeting to facilitate the end-user with the guaranteed facilities [11]. Many researchers have widely deployed the ring topology in OMANs due to its self-healing characteristics and reliability for access networks [12,13,14]. X. Li et al. deployed a dual fiber ring structure for protection measurements and multiple transmitters at the optical line terminal side (OLT) [15]. Similarly, a ring-based OMAN network is deployed with two OLTs in [16] for protection purposes.

The number of frequencies in an OMAN is rising in parallel with the country’s increasing need for high-speed data transmission. Because of this, many light sources are needed to accommodate many users [15]. The OLT’s price tag is critical in determining the final bill. It is possible to generate a wide range of frequencies from a single laser source using an optical multicarrier (OMC) generator [17,18,19]. The cascaded arrangement of a different or a single modulator is one of several recent ways of generating OMCs [20,21,22,23]. The OMC is used in the optical access network [22,24,25], free space optics [26], in the OMAN [17,27], and for other applications [28]. By boosting efficiency and simplifying maintenance, the OMC generators deployed in an OMAN can efficiently deliver a variety of frequencies. A spectrum-efficient network with an add–drop multiplexer and a centralized OMC source was utilized for the OMAN. Similarly, the carrier wavelength reuse concept was presented using an OMC-based ring network and a reconfigurable optical add–drop multiplexer [29].

In the provided references, some papers have proposed OMC-based sources as the best solution for an OMAN but did not provide detailed proposals of their OLTs [27,30,31]. In the OMAN, each user needs a specific wavelength, which increases the OLT’s cost. A similar add–drop multiplexer has been used on the ONU side, but it is unable to drop many frequencies at once and is hence unable to handle the OMC source [31,32,33]. In the OMAN, in a core network, a ring architecture is an ideal topology for connecting different nodes, and an OLT is the main source for data handling/transmission to the ONUs. Therefore, for a ring-based architecture, two or more OLTs are tested in case a fiber failure occurs in the core network [16]. Hence, deploying more OLTs increases network costs. Most importantly, the data rate in the cited work is not sufficiently high to fulfill the demands of applications for the next-generation access network. 

OpEx and CapEx constraints and the development of metro network traffic have led to a search for architectures with reduced energy consumption, smaller footprints, and cheaper costs. For this reason, a centralized OLT in the OMAN using WDM technology is configured in a ring architecture at the feeder fibers. At the same time, star topology is set up at the user end. Deploying OFCs in an OMAN is important for enhancing data transmission by providing high bandwidth to each user with high-frequency spacing. This could ensure a high data rate transmission efficiently with superb scalability, provide low latency, and fulfil the urban network demands. 

An OMCG-based OLT, which can efficiently transmit 20 Gbps duo-binary modulated data at a frequency spacing of 20 GHz, is presented in this study. At each OMAN remote node (RN), optical switches (OSws) can support downlink and uplink transmission. Our OMC spectrum distribution divides the entire wavelengths into six RNs, with ten wavelengths each. Depending on the end-user’s location, this system can increase or decrease the number of RNs and ONUs. At each RN, the ONUs are connected through star-topology distribution cables. Overall, 60 ONUs are connected to the central office, enabling a broadcast of 1200 Gpbs, with 20 Gpbs being allocated to each user at varying distances. WDM-PON assigns each subscriber a distinct wavelength. By eliminating ROADMs, OADMs, and various light sources, a cost-effective and efficient OMAN is presented and supported by the results. The main contributions of this paper are summarized here for easy understanding by the readers:

- This study proposes a new central office for an OMAN based on an OMC generator, focusing on affordability and an efficient switching system.
- The OMCG is based on a cascaded configuration of a phase modulator (PM) and two Mach Zehnder modulators (MZMs), driven by an RF signal controlling the carriers’ frequency spacing. Over sixty frequencies are generated with a high tone-to-noise ratio (TNR) of 40 dB and minimal amplitude difference among the generated carriers.
- An efficient switching mechanism is introduced in the proposed OMAN to handle the transmissions during the fiber failures in the main core fiber in the ring architecture. This failure could be a single fiber failure or a multistage fiber failure, which are also considered and presented for proper maintenance/management of the transmission. The proposed CO design can cope with different scenario-based problems without compromising the transmission quality.

## 2. Architecture of the Proposed Optical Metro Access Network

**Figure 1:**
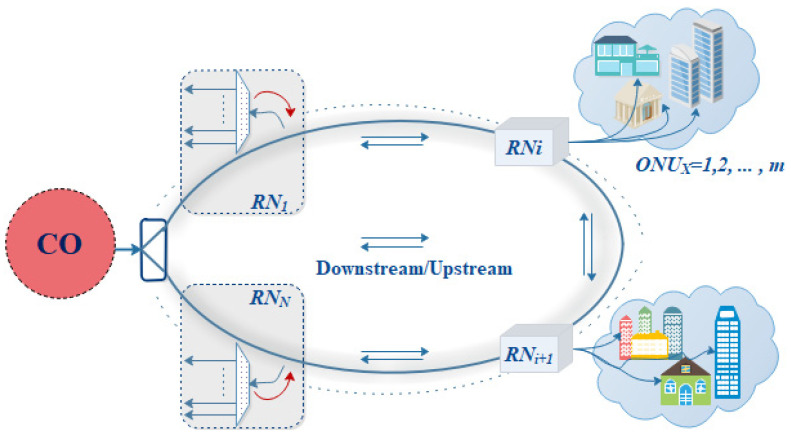
The proposed metro access network. CO: central office; RN: remote node; ONU: optical network unit.

Figure 1 shows the OMAN’s dual-fiber ring construction. The metro access network consists of one or two central offices and M RNs [9,15,16,29,30], with a ring feeder fiber design. When building an OMAN, redundant fibers are buried [14]. A feeder fiber is utilized for network protection; when one fiber is broken, transmission can be shifted to the second fiber. Dormant access ring fibers can be utilized for protection. A dual-fiber ring connects the CO and RNs in this network. One central office and six RNs can distribute multiple wavelengths to access nodes. It can increase (RNs→N) based on the access node location. Providing multiple wavelengths (λ1→λm) can also enhance ONUs. In the CO, the OMCG OLT offers 61 stable and healthy frequency tones.

### 2.1. Schematic of the CO

**Figure 2:**
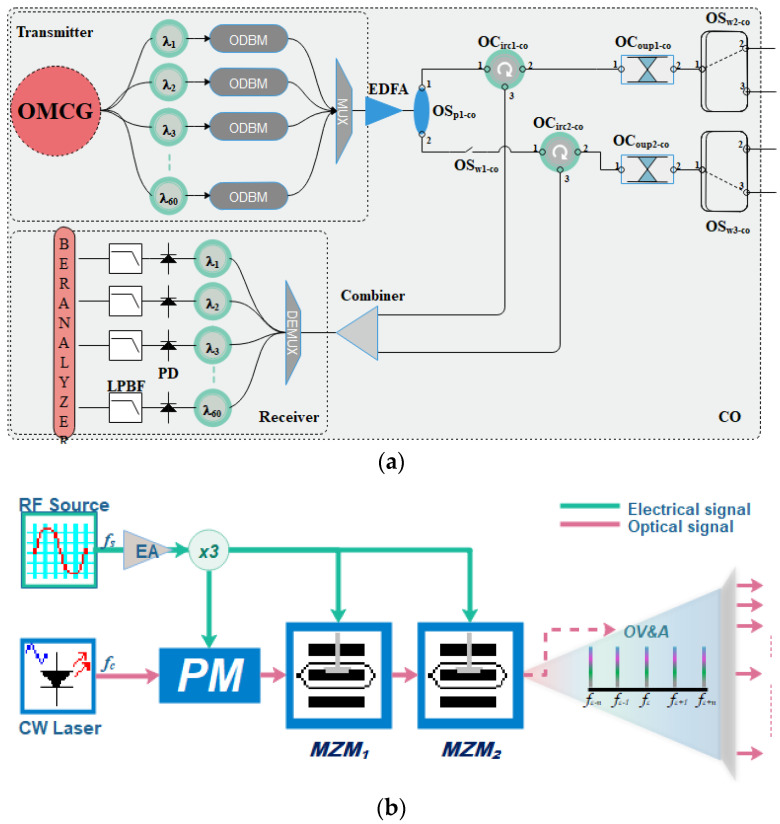
(**a**) Schematic diagram of the proposed central office. OMCG: optical multicarrier generator; ODBM: optical duo-binary modulator; EDFA: erbium doped fiber amplifier; optical switch: optical switch; OC: optical coupler; PD: photodetector; LPBF: low bandpass Bessel filter (**b**) Schematic of the proposed optical multicarrier generator. EA: electrical amplifier; PM: phase modulator; MZM: Mach–Zehnder Modulator; OV and A: optical visualizer and analyzer.

The schematic diagram of the proposed CO is shown in Figure 2a. It is usually more favorable for the OMAN to have a great number of frequency channels. This can be achieved by either using laser arrays or frequency combs. We have utilized an OMC generation scheme, shown in Figure 2b, to provide several carriers to facilitate over 60 end-users with high data rates. 

The proposed technique in this study can handle higher data rates with increased frequency spacing and practically double the bandwidth. The proposed OLT, placed at the CO of the metro network, is shown in Figure 2a. It essentially eliminates the need for a single laser diode per single subscriber. The end-face of the multicarrier generator is connected to a 1xN optical fork (a fork with *N* outputs) arrangement, followed by a set of optical filters to separate the frequencies. The filtered carriers are injected into an optical duo-binary modulator ~20 Gbps (ODBM). An optical multiplexer combines the modulated signals into one (MUX). A 1 × 2 optical splitter (OSp1-co) amplifies and splits the multiplexed signal, which is then followed by two optical circulators (OCircs-co), optical couplers (OCoups-co), and optical switches (OSws-co). The OSp1-co’s first port is coupled to the OCirc1-co’s input port1. The OCirc1-co’s port 2 is linked to the OCoup1-co’s port 1. To address the demand for network expansion, the OSp1-co’s second port is connected to an optical switch (OSw1-co). Following the switch is OCirc2-co, which has the same structure as OCirc1-co. The OCoup1-co and OCoup2-co end-ports are connected to OSw2-co and OSw3-co, which direct the signal to either the operating mode fiber or the protection fiber. The protection mechanism section explains the needs and working mechanism of OSw2-co and OSw3-co. This arrangement of OCirc-co, OCoup-co, and OSw-co is for normal working conditions, and one is kept reserved for the protection mode for the primary feeder fiber, both for uplink and downlink transmission. The switch manages the uplink and downlink transmissions in normal conditions or when a fault is detected, it switches the transmission into the reserved/protected fiber cable.

Similarly, the CO contains another section of uplink transmission after receiving the data during the normal operation via OSw2-co or during the protection mode through OSw3-co. The data are passed from the OCoups-co to the OCircs-co via port2 and forwarded to the power combiner via port 3 of the OCircs-co. The outputs of these two OCircs-co are combined to receive the signal from both modes directly. The receiver section is further discussed in the coming sections.

### 2.2. The Principal of the OMC Generator

The OMC generator is based on the cascaded configuration of a single phase modulator (PM) and two Mach Zehnder modulators (MZMs). This proposed technique uses electro-optic modulators driven by an RF source that produces a multiharmonic signal, separated by a fixed frequency termed frequency spacing that can be modified using the RF signal. The optical input of the PM is connected to the CW laser source, whereby the first MZM (MZM1)’s optical input is driven by the PM’s output and the second MZM (MZM2) by the output of the first MZM. 

The phase modulator (PM) changes the injected optical signal in response to the provided amplified RF signal. Consider the optical continuous wave (CW) with electric field *E*_0_ and angular frequency *ω*_0_. The CW’s signal before entering the PM can be given as $E_{cw}(t)=E_{0}e^{j\omega_{0}t}$, where *E*_0_ represents the amplitude, and ω_0_ represents the angular frequency of the laser. The RF amplified sinusoidal signal can be described as $V_{RF}(t)=V_{\max}(\cos(\omega_{RF}t+\phi )$, where *V_max_* and $\omega_{RF}$ represent the amplitude and angular frequency of the RF source. The phase modulator produces a phase shift that is proportional to the provided RF signal, which can be given as *ϕ*. Therefore, the phase-modulated optical signal can be given as

(1) $$E_{PM}(t)=E_{cw}(t)\exp(jG\beta \sin(\omega_{RF}t+\phi ))$$

where *G* is the gain in the RF signal, and we will consider it a constant value ~1 for ease of understanding the further equations, *β* is the modulation index of the PM, *ϕ* is the phase of the RF signal, and *t* represents the time. Therefore, the term $\beta \sin(\omega_{RF}+\phi )$ represents the phase modulation introduced by the PM. After expanding the above equation, it can be given as

(2) $$E_{PM}(t)=E_{0}\exp(j\omega_{0}t)\exp(j\beta \sin(\omega_{RF}t+\phi ))$$

**Figure 3:**
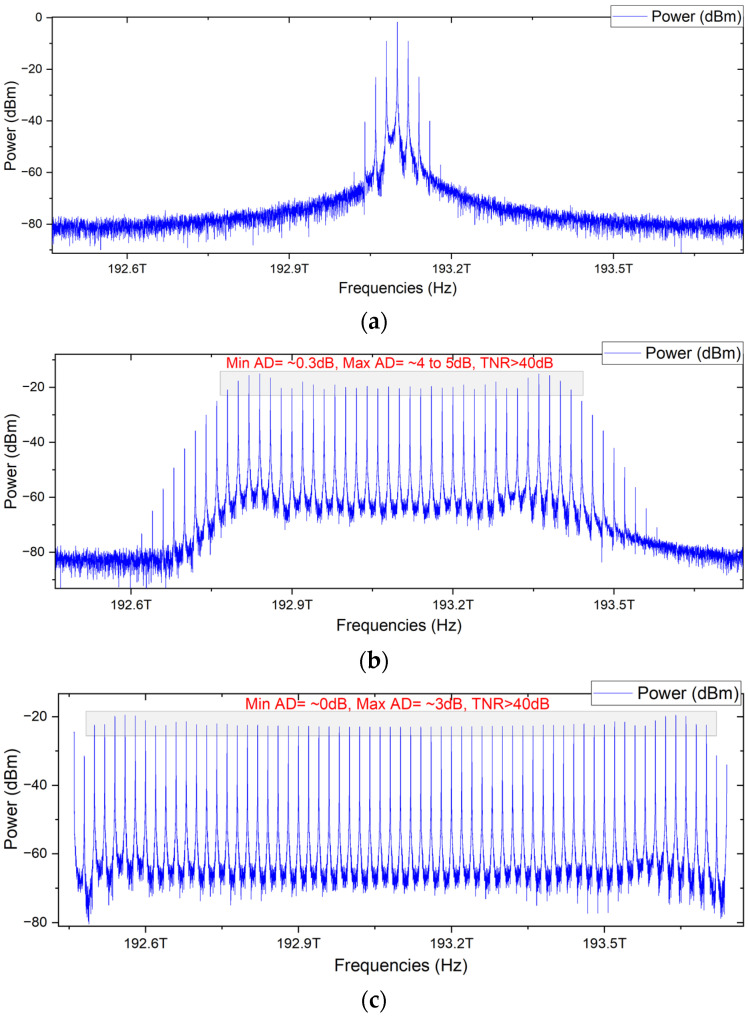
Generation of OMC using three modulators; (**a**) output of the phase modulator; (**b**) output of MZM1; (**c**) output of MZM2.

Equation (2) can be expanded further by using the well-known Jacobi–Anger expansion to express the phase-modulated signal in terms of the Bessel functions, as given below:

(3) $$E_{PM}(t)=E_{0}\exp(j\omega_{0}t)\sum_{n=-\infty }^{n=\infty }J_{n}(\beta )\exp(jn(\omega_{RF}t+\phi ))$$

where *J_n_* is the *n*-th order of the Bessel function of the first kind. This expression shows the creation of sidebands with frequencies $\omega_{0}\pm n\omega_{RF}$, with their amplitude being determined by the Bessel function $J_{n}(\beta )$, and $\omega_{RF}$ represents the frequency spacing between the comb lines. This equation captures the essence of phase modulation under the impact of an RF signal. The Bessel functions represent the energy distribution created due to modulation among various sidebands. The output of the PM is shown in Figure 3a. The output of the PM is connected in cascade to the first MZM. It modulates the intensity of the optical signal whose output can be given as

(4) $$\begin{array}{ll} E_{MZM1}(t) & =E_{PM}(t).[1+m\cos(\omega_{RF}t)]\\ & =E_{0}\exp(j\omega_{0}t)\sum_{n=-\infty }^{n=\infty }J_{n}(\beta )\exp(jn(\omega_{RF}t+\phi )).[1+m\cos(\omega_{RF}t)] \end{array}$$

As $\cos(\omega_{RF}t)=\frac{1}{2}[\exp(j\omega_{RF}t)+\exp(-j\omega_{RF}t)]$, therefore, after putting this in the above Equation (4), it becomes

(5) $$\begin{array}{r} E_{MZM1}(t)=E_{0}\exp(j\omega_{0}t)\sum_{n=-\infty }^{n=\infty }J_{n}(\beta )\exp(jn(\omega_{RF}t+\phi )).\\ \left[1+\frac{m}{2}\{\exp(j\omega_{RF}t)+\exp(-j\omega_{RF}t)\}\right] \end{array}$$

Here, *m* shows the modulation depth of the MZM that controls the additional sidebands generated by the MZM. The above expression shows a sum of frequency components that is generated by the PM and then further modulated by the first MZM, where every term corresponds to a separate frequency tone at both sides of the central frequency ~$j_{0}(\beta )$. This comb is practically finite, because the Bessel function decreases with increasing of the “*n*”. After passing this modulated OFC through the second MZM, more carriers are added to the OFC, which can be presented as

(6) $$\begin{array}{ll} E_{MZM1}(t) & =E_{0}\exp(j\omega_{0}t)\sum_{n=-\infty }^{n=\infty }J_{n}(\beta )\exp(jn(\omega_{RF}t+\phi )).\\ & \left[1+\frac{m}{2}\{\exp(j\omega_{RF}t)+\exp(-j\omega_{RF}t)\}\right].\left[1+\frac{m^{\prime }}{2}\{\exp(j\omega_{RF}t)+\exp(-j\omega_{RF}t)\}\right] \end{array}$$

If both MZMs are identical, then it can be given as

(7) $$\begin{array}{r} E_{MZM2}(t)=E_{0}\exp(j\omega_{0}t)\sum_{n=-\infty }^{n=\infty }J_{n}(\beta )\exp(jn(\omega_{RF}t+\phi )).\\ {\left[1+\frac{m}{2}\{\exp(j\omega_{RF}t)+\exp(-j\omega_{RF}t)\}\right]}^{2} \end{array}$$

This equation is mathematically complex because of the double modulation effect, where each frequency tone from the first MZM is further increased. Hence, we developed a large bandwidth in the proposed scheme. 

An amplified 1 × 3 RF signal is used to modify the electrical inputs of these modulators. The RF source frequency is kept constant at 20 GHz, the amplitude and DC biasing of the RF are 1 a.u., and the sample rate is 1.28 × 10^12^. The frequency of the CW laser is 193.1 THz as a center frequency, with a linewidth of 10 MHz and dynamic noise of 3 dB. The PM converts the signal into a wideband signal composed of ultra-short optical pulses that are broadened by the MZM1. The output signal is further expanded by employing the MZM2, which achieves over 60 ultra-flat optical carriers. Figure 3 depicts the output of the three modulators. The resulting optical spectrum, shown in the image, has a tone-to-noise ratio of more than 40 dB and amplitude differences of 0–3 dB. The scheme has a total bandwidth of 1.2 THz. The proposed network for OFC generation has successfully generated over sixty carriers which is higher than or equal in numbers to most of the cited works [19,20,25,28,34].

## 3. Downlink and Uplink Transmission Operations

**Figure 4:**
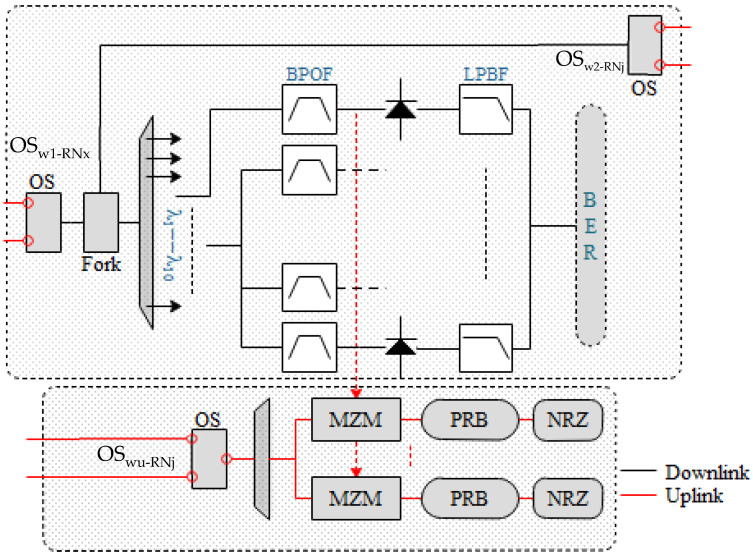
Schematic diagram of the RN + ONU.

During the regular mode operation, the downlink and uplink data are transmitted in the feeder fibers (working fibers) from the CO to RNs and vice versa. The CO contains an OMC source as an OLT that generates X number of frequencies, utilized for downlink transmission, which can be received by Y number of receivers (X = Y) back into the CO after an uplink transmission. The X number of transmitted wavelengths is received by the M number of RNs, where each RN passes Z number of frequencies. A DeMUX can separate the desired tones for the dedicated number of ONUs. As demonstrated in Figure 2a, port 1 of the OSw1-co is connected to the working fiber. The schematic of RN and ONU is shown in Figure 4, where OSw1-RNx followed by a 1×2o-fork receives the signal. The 1×2o-fork performs the following two tasks: (i) it extends the traffic towards the end-users through 1xNo-fork, or by using DeMUX to separate the frequency tones, where N represents the number of users connected to the fork/DeMUX, and (ii) it extends the signal from output port 2 towards the second switch (OSw2-RNj), which extends the fiber in the clockwise direction towards the next RN (OSw-RNj1), where the same procedure is repeated and a ring network is formed. Consequently, if “M” RNs are connected in the ring, and at each RN, 1:No-forks are connected across the ring, the total number of subscribers in the network can be given by M × N. The end-face of each 1:No-fork or the DeMUX terminates in the subscriber premises through a distribution fiber (DF). The DF is connected to a bandpass filter that works as a DeMUX arrangement and recovers the desired carrier frequency for the intended subscriber, which is split in two by a 1 × 2 OSp; half of it is used for demodulating the signal. In contrast, half of the signal’s power is used for colorless uplink transmission. 

In the proposed setup, the multiplexed signal that is transmitted from the CO is received by six RNs. Therefore, the overall spectrum of 1.2 THz is divided into six groups of frequencies with a range from 192.50 THz to 193.70 THz. The frequency range dedicated to the RN1 is from 192.52 to 192.70 THz. Similarly, RN2 is from 192.72 to 192.90 THz, and so on. At each RN, ten tones are dedicated to the end-users. The summary of the frequency distributions among the different RNs is given in Table 1.

**Figure 5:**
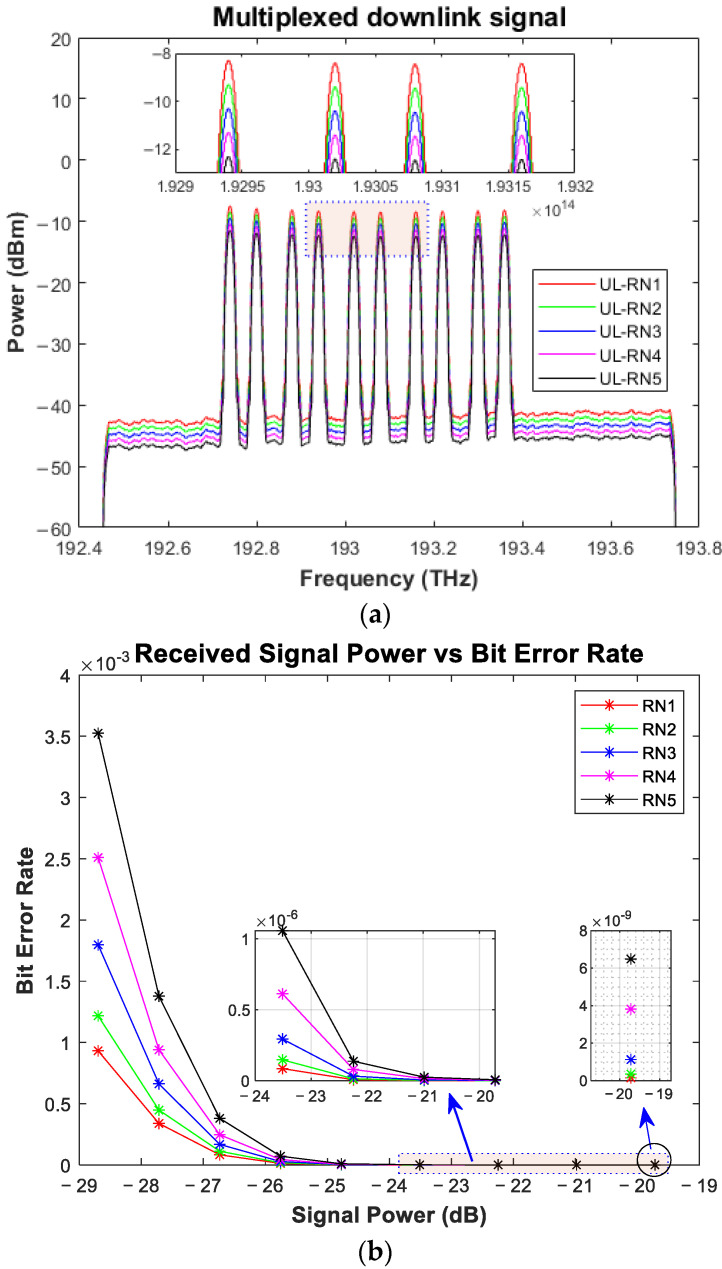
(**a**) The multiplexed signal received at each RN: (**b**) bit error rate (BER) vs. received signal power.

For downlink transmission among the six RNs, we simulated five RNs, and for each RN, two frequencies were selected for carrying the 20 Gbps ODBM-based modulated data. As mentioned in Table 1, 60 subscribers can be facilitated at six different RNs, where each RN is at the span of 5 Km feeder fiber, while each user is at a distance of 2 km from the RN. In this huge network, we have provided the simulation results for two users per RN for downlink and uplink transmission. The multiplexed signal received at 5 km (at RN1), 10 km (at RN2), and up to 25 km (at RN5), each with a fiber span of 5 km, is demonstrated in Figure 5a, which shows the drop/loss of power from one ONU to the other. The power loss across each RN can be observed clearly in the figure. To test the applicability of the scheme, a variable optical attenuator (VOA) is used at the output of the OS_w1-co_ and OS_w2-co_. The VOA attenuates the signal’s power level and distorts the signal, and we can calculate the desired BER values. The value of the VOA is set in an iterative manner, where the min value is 0, and the maximum is restricted to 9. Each iteration advances the value by an addition of 1. The simulation parameters are listed in Table 2.

The achieved low bit error rate of 10^−13^ to 10^−3^ is shown in Figure 5b, which shows a min. BER of approximately 10^−13^ for 20 Gbps downlink transmission. Hence, the scheme guarantees efficient performance for 20 Gbps downlink transmission. The graph shows the power loss across the channel and other passive components. 

**Figure 6:**
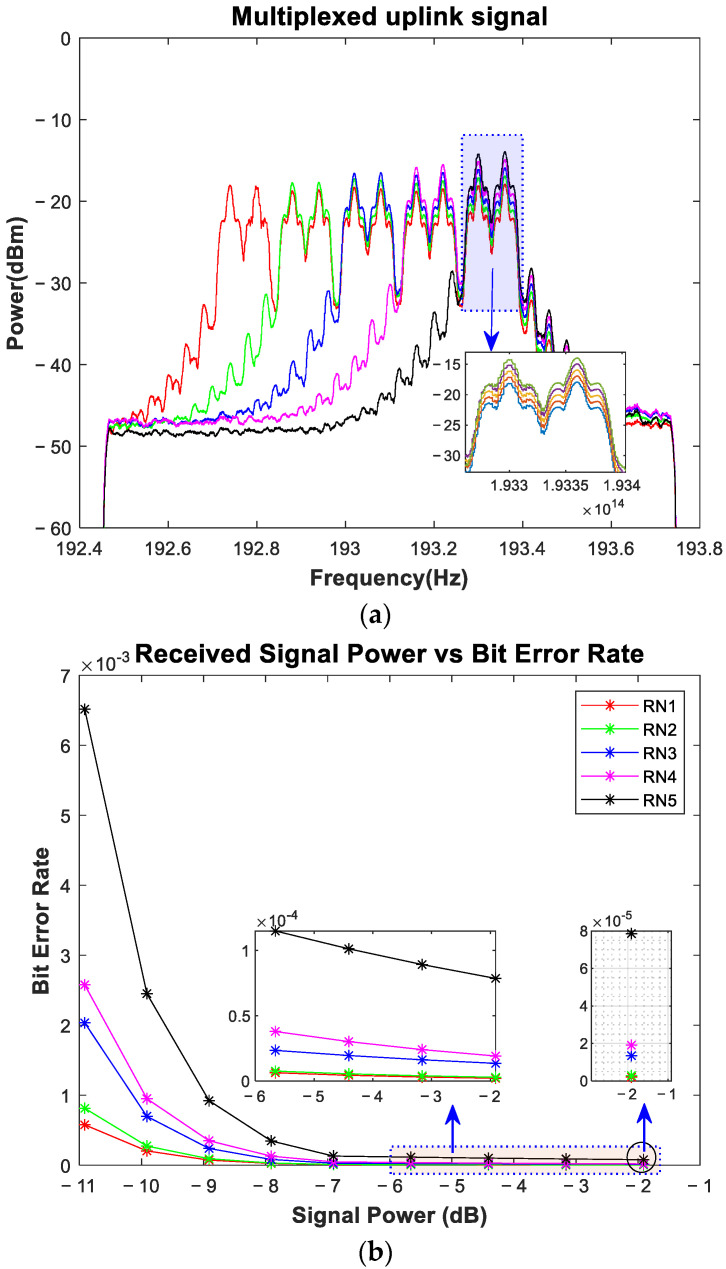
(**a**) The multiplexed uplink signal received from five RNs: (**b**) the BER vs. the received signal power [data rate = 10 Gbps].

Upon receiving the signal at *OS_w1-RNj_*, as shown in Figure 4, which is split after the identification via an optical filter, part of it is reused for uplink transmission. The signal is identified using a Gaussian optical filter and fed into an intensity modulation on-off keying (IM-OOK) for data modulation. For uplink transmission, the data rate of the simulation environment is reduced to 10 GHz. The use of a duobinary modulation scheme in the downlink transmission proved to be useful to re-modulate the downstream signal into the OOK upstream signal at the RN [35]. A symmetrical operation at 10 Gbps over a 25 km reach has already been proposed and demonstrated experimentally [36]. Even a symmetric 10 Gbps WDM-PON has been proven to reach distances of 80 km without using any remote amplification [37]. After modulation, the signals of both ONUs are combined and sent back to the optical fiber. Before injecting the signal into the fiber, *OS_wu-RNj_* is used to direct the signal to the working fiber or protection fiber. The OSw1-co and OSw2-co are utilized at the central office to identify the signal mode. If the signal is received from the working fiber, the *OS_w1-co_* directs the signal to the *OC_oup1-co_*, which is then forwarded to the *OC_irc1-co_*. If the signal is received from the protection fiber, the *OS_w2-co_* directs the signal to the *OC_oup2-co_*, which is then forwarded to the *OC_irc2-co_*. The output of both circulators is combined using an ideal Optical Combiner, and the output is fed into a 1 × 10 WDM De-MUX. A Gaussian optical filter is utilized at the De-MUX to identify each wavelength. After wavelength identification, a direct detection optical receiver (DDOR) receives the signal. The DDOR is based on a PIN photodiode and a low-pass Bessel filter, and the results are calculated using BER spectrum analyzers. The received channels promise a good performance, and the results can be seen in Figure 6a, where the multiplexed signal shows a continuous downfall in the received optical power of around 1 dBm. The same VOA is used to test the performance of the scheme. When the value of the VOA is at its minimum, the minimum BER is exceptionally low, whereas the scheme offers a minimum BER of slightly below 10 × 10^−3^ for the high attenuation value, as shown in Figure 6b, which greatly supports the applicability of the proposed scheme.

## 4. Protection Mechanism

**Figure 7:**
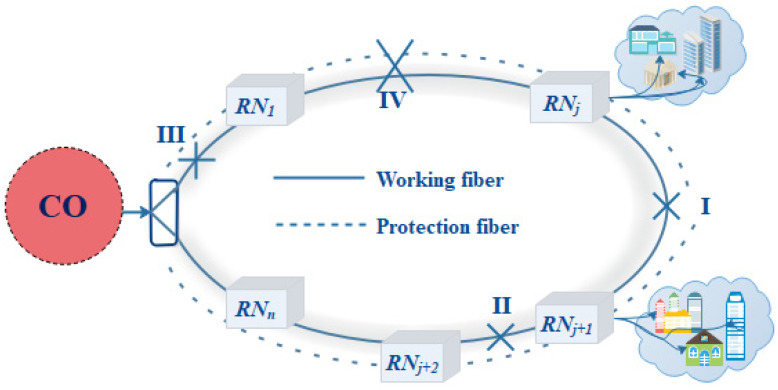
Fiber failures at different positions.

**Figure 8:**
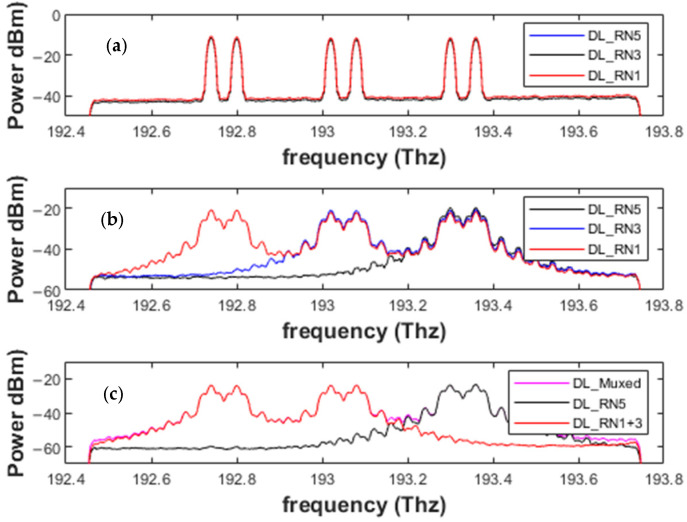
Simulation results: (**a**) multiplexed downlink signal at each RN, (**b**) received signal from each RN during class-A failures, and (**c**) received signal from each RN during a class-B failure.

The protection mechanism of the proposed scheme can be characterized by two categories: fault-A and fault-B. The fault-A protection mechanism can be considered when a single working feeder fiber fails between any two RNs, i.e., *RN_j_* and *RN_j+1_*; then, the *OS_w1-co_* or the *OS_w2-RNj_ at RN_j_* transfers the load to the second port that is connected with the protection fiber. Similarly, the optical switch at RNj+1 transfers the load to the switch’s second port, which accepts the data from the protection fiber. Therefore, normal operation can be easily restored after repairing the working feeder fiber by utilizing the protection fiber. This platform can work even for numerous single-feeder fiber faults, as it easily transfers the transmissions to the protected fiber. As shown in Figure 7, three cases, I, II, and III, show the faults at three single feeder fibers at different positions. The simulation results of these cases can be studied in Figure 8. The receiving powers across the downlink transmissions show us the power loss during the transmission and during the fiber failure cases.

The fault-B protection mechanism is used when the feeder fiber fails and *OS_w2-co/RNj_* cannot restore normal operation. In the situation that occurred at I and II, the fault protection mechanism works as we described earlier. For the fault at IV, the state of the binary switch *OS_w1-co_* is changed before the *OC_irc2-co_* is turned on, and the signal is transmitted across the *OC_irc_*. This signal is connected to the *OS_w2-co_*. Here, the direction of the signal is changed, and the signal at *OS_w1-co_* is directly received by the RN1, whereas the RN1 and RN5 are no longer connected due to a failure of the feeder and protection fiber, and the *OS_w2-co_* changes the flow and transmits the signal to RN5. The working mechanism of the upload transmission is the same for RN1; however, the uplink signal generated by RN5 can no longer connect to RN1. Therefore, the uplink signal is also received by the *OS_w2-co_*. All the switches in the CO (OS_w1,2,3-co_) and ONUs realize the working conditions in normal and protection mode. 

Similarly, the proposed model can also work in the dual feeder fiber failure, i.e., if both the normal mode fiber and the protection fibers fails, as shown in Figure 7 by IV between the RN1 and RNj. In this scenario, the switch in the CO (OSCO-1) can manage both the downlink and uplink transmission, and both the branches will work in the two tree networks for the time being, until the repairing of the feeder fibers occurs. 

## 5. Conclusions

This paper introduces a centralized single OLT to support many users in a WDM ring architecture-based OMAN. A traditional OLT of the OMAN is replaced with a cost-effective OMC source that offers numerous frequency tones to carry the bidirectional information across different spans of fibers. These fibers are connected in a ring architecture with different RNs, and ten ONUs can be connected to each RN based on the star topology. At each RN, part of the downlink signal, which can be considered colorless, is utilized for uplink transmission. The OMC source generates more than sixty comb lines with measurable parameters, making it more suitable to deploy in an OMAN that is meant for entertaining many subscribers while facilitating heavy data rate support. Across each carrier of the generated OFC, 20 Gbps data are transmitted based on optical duobinary modulation. At the ONU side, VOAs are utilized to calculate the BERs of the received signal, which ranges from 10^−3^ to 10^−13^ for the downlink transmission. Similarly, an OOK is utilized for the uplink transmission to test the feasibility of the proposed OFC in the OMAN system. The BER during the uplink transmission ranges from 10^−6^ to 10^−14^. Furthermore, the detailed analyses are discussed and explained with their respective results for the various cases of main optical fiber failures in the optical metro access network, along with the system’s robust switching mechanism.

## Figures and Tables

**Table 1:** Summary of the frequency distribution.

RNX	Frequencies per RN	Frequency Ranges (20 GHz Frequency Spacing)
RN1	F_01_ to F _10_	192.52 THz→192.7 THz
RN2	F_11_ to F_20_	192.72 THz→192.9 THz
RN3	F_21_ to F_30_	192.92 THz→193.1 THz
RN4	F_31_ to F_40_	193.12 THz→193.3 THz
RN5	F_41_ to F_50_	193.32 THz→193.5 THz
RN6	F_51_ to F_60_	193.52 THz→193.7 THz

**Table 2:** Simulation parameters.

Parameter	Value	Parameter	Value
Global Parameters		Optical Fiber	
Bit rate	20 × 10^9^ bit/s	Reference wavelength	1552 nm
Sequence length	256 bits	Length	5 km
Samples per bit	128	Attenuation	0.2 dB/km
Symbol rate	10 × 10^9^	Dispersion	16.75 ps/nm/km
CW Laser		Dispersion slope	0.075 ps/nm^2^/km
Frequency	193.1 THz	Differential group delay	0.2 ps/km
Power	5 dBm	Effective area	80 um^2^
Linewidth	0.1 MHz	Fract. Raman contribution	0.18

## Data Availability

Data are contained within the article.
